# Influence of acellular dermal matrix on peri-implant soft tissue phenotype and marginal bone loss following immediate implant placement in the anterior maxilla: a randomized controlled clinical trial

**DOI:** 10.1186/s12903-026-10072-4

**Published:** 2026-11-04

**Authors:** Mai Ahmed Haggag, Mohammed Feheid Alrawooji, Amira M.M. Attia

**Affiliations:** Faculty of Dentistry, Mansoura University, Mansoura, Egypt

**Keywords:** Acellular dermal matrix, Immediate dental implants, Soft tissue augmentation, Peri-implant phenotype, Marginal bone loss

## Abstract

**Background:**

Immediate implant placement in the anterior maxilla is associated with dimensional changes in both hard and soft tissues, which may compromise esthetic outcomes. This randomized controlled clinical trial aimed to evaluate the effect of acellular dermal matrix (ADM) on peri-implant soft tissue phenotype and marginal bone loss following immediate implant placement.

**Methods:**

Patients requiring replacement of non-restorable maxillary anterior teeth were randomly allocated into two equal groups. The control group received high-density polytetrafluoroethylene (d-PTFE) membrane coverage, while the study group received ADM. Clinical parameters, including keratinized mucosal width (KMW), mucosal thickness (MT), and supracrestal tissue height (STH), were assessed at 6 months. Plaque accumulation and bleeding were monitored using modified sulcus bleeding index (mSBI) and plaque index (PI) at 1, 2, and 3 months after prosthetic loading. Implant stability was measured at placement and after 6 months. Radiographic outcomes, including marginal bone loss (MBL) and bone width (BW), were assessed using fused CBCT images at baseline and 6 months. In the study group, ADM was inserted into facial and palatal pouches. The primary outcome was peri-implant soft-tissue phenotype at 6 months, assessed by KMW, MT, and STH.

**Results:**

Thirty-three patients were assessed for eligibility, and sixteen patients were randomly allocated into two equal groups (*n* = 8 per group). All implants survived without complications during follow-up. At 6 months, the study group demonstrated significantly greater keratinized mucosal width (5.9 ± 1.0 vs. 3.3 ± 0.6 mm, *p* < 0.001), mucosal thickness (1.8 ± 0.3 vs. 0.8 ± 0.3 mm, *p* < 0.001), and supracrestal tissue height (1.2 ± 0.3 vs. 0.8 ± 0.3 mm, *p* = 0.012) than the control group. No significant differences between groups were observed in mSBI or PI. Implant stability increased significantly in both groups, with higher values in the ADM group at 6 months (*p* = 0.010). The study group also exhibited significantly less marginal bone loss, whereas horizontal bone width reduction was comparable between groups.

**Conclusion:**

ADM was associated with improved peri-implant soft tissue phenotype and reduced marginal bone loss compared with d-PTFE membranes while maintaining comparable peri-implant health. These findings support the potential use of ADM during immediate implant placement in the anterior maxilla.

**Trial registration:**

The study was retrospectively registered and listed on www.clinicaltrials.gov with registration number (NCT07573020) on 07-05-2026.

## Background

Dental implant therapy has evolved from a primary focus on osseointegration toward achieving optimal functional and esthetic outcomes, particularly in the anterior maxilla where esthetic demands are high. Immediate implant placement, defined as implant insertion into a fresh extraction socket, has gained popularity due to reduced treatment time and fewer surgical interventions [1].

Despite survival rates comparable to delayed placement protocols, immediate implant placement remains technique-sensitive and highly dependent on local anatomical conditions. Following tooth extraction, physiological bone remodeling leads to dimensional changes of the alveolar ridge, with pronounced resorption occurring at the buccal aspect due to the thin facial bone plate, which is often less than 1 mm thick. These changes may compromise both hard and soft tissue architecture, negatively affecting esthetic outcomes [2–4]. 

The peri-implant soft tissue phenotype plays a critical role in long-term stability and esthetics. A thin gingival phenotype is associated with an increased risk of mucosal recession and marginal bone loss, whereas a thicker phenotype provides improved resistance to mechanical and inflammatory challenges. Therefore, augmentation of peri-implant soft tissues has been proposed to enhance tissue stability and esthetic integration [5, 6]. 

Autogenous connective tissue graft (CTG) is considered the reference approach for peri-implant soft tissue augmentation due to its predictable ability to increase peri-implant mucosal thickness and improve soft tissue stability and esthetic outcomes [7, 8]. However, CTG harvesting requires a second surgical site, which may increase postoperative morbidity, surgical time, and patient discomfort, leading to the development of alternative biomaterials such as acellular dermal matrix [9].

ADM is a biologically derived allograft that serves as a scaffold for cellular repopulation and revascularization, promoting soft tissue augmentation without the need for a second surgical donor site. In contrast, d-PTFE membranes act as physical barriers that prevent epithelial migration but lack biological activity [10, 11]. 

While both approaches are used in clinical practice, limited evidence exists comparing their effectiveness in modifying peri-implant soft tissue phenotype and preserving peri-implant hard tissues in immediate implant placement [12]. 

Recent systematic reviews indicate that peri-implant soft-tissue augmentation can increase mucosal thickness and support tissue stability. However, evidence specifically addressing ADM during immediate implant placement remains limited, and direct comparisons between ADM and d-PTFE socket coverage are scarce [6, 13]. 

Therefore, this randomized controlled clinical trial aimed to compare the effect of ADM and d-PTFE membrane on peri-implant soft tissue phenotype following immediate implant placement in the anterior maxilla. The primary endpoint was peri-implant soft tissue phenotype, assessed by keratinized mucosal width, mucosal thickness, and supracrestal tissue height at 6 months. Secondary endpoints included marginal bone loss, bone width, implant stability, and peri-implant clinical indices. The null hypothesis was that there would be no significant difference in peri-implant soft tissue phenotype between the ADM and d-PTFE membrane groups following immediate implant placement in the anterior maxilla.

## Patients and methods

### Ethical statement

This study was designed as a parallel-group randomized controlled clinical trial conducted at the Faculty of Dentistry, Mansoura University, Egypt. Ethical approval was obtained from the institutional Research Ethics Committee (No. R.26.04.114). All participants provided written informed consent prior to enrollment. The study was conducted in accordance with the Declaration of Helsinki and reported following the CONSORT guidelines [14]. Written informed consent was obtained from all participants prior to enrollment. The study was listed on www.clinicaltrials.gov with registration number (NCT07573020) on 07-05-2026.

### Patient selection

A total of 16 patients requiring replacement of non-restorable maxillary anterior teeth were recruited from the outpatient clinic of the Oral and Maxillofacial Surgery Department.

#### Inclusion criteria

Patients aged 18–45 years requiring immediate replacement of a single non-restorable maxillary anterior tooth. Only intact extraction sockets (Type I socket according to the Elian classification) [15] with preserved labial and palatal socket walls, sufficient apical bone to achieve primary implant stability, good oral hygiene, adequate interarch space [16], Only sites exhibiting a thick gingival phenotype, as determined during the preoperative clinical examination and the ability to comply with the study protocol were included.

#### Exclusion criteria

Any local pathological condition that contraindicates implant placement, pregnancy, smoking, medication that affects bone healing, psychological problems, radiation therapy to the head and neck area in the past 12 months [17]. 

### The study design

Randomized Controlled Clinical Trial.

#### Hypothesis

Null hypothesis (H_0_):

here would be no statistically significant difference between the ADM group and the d-PTFE membrane group with respect to the peri-implant soft tissue phenotype following immediate implant placement in the anterior maxilla.

### Sample size calculation

Sample size was calculated a priori using G*Power software (version 3.1.9.7). A two-sided Mann-Whitney U-test was specified, assuming a Laplace distribution, a significance level of α = 0.05, an allocation ratio of 1:1, and an anticipated standardized effect size of 1.40. Under these assumptions, a sample size of 8 participants in Acellular Dermal Matrix group and 8 participants in d-PTFE group was estimated to provide 90.59% statistical power to reject the null hypothesis. The anticipated magnitude of the treatment difference was informed by the available literature on acellular dermal matrix applications in oral soft tissue augmentation, including the clinical evidence summarized by Dadlani, 2021 [18]. 

### Randomization, allocation concealment, and blinding

Participants were randomly allocated in a 1:1 ratio to either the ADM group or the d-PTFE membrane group using a randomized block procedure. A block size of 4 was selected to maintain balanced allocation between the two treatment groups throughout the enrollment period. Allocation concealment was maintained during patient enrollment. The surgeon was aware of the assigned intervention due to the nature of the surgical procedures; however, the clinical outcome assessor and radiographic evaluator were blinded to treatment allocation during outcome assessment.

#### Patients’allocation

All patients underwent atraumatic tooth extraction followed by immediate implant placement.

- Control group: Implant placement with socket coverage using d-PTFE membrane.
- Study group: Implant placement with ADM graft inserted into labial and palatal pouches.

### Surgical protocol

#### Preoperative phase

**Fig. 1:**
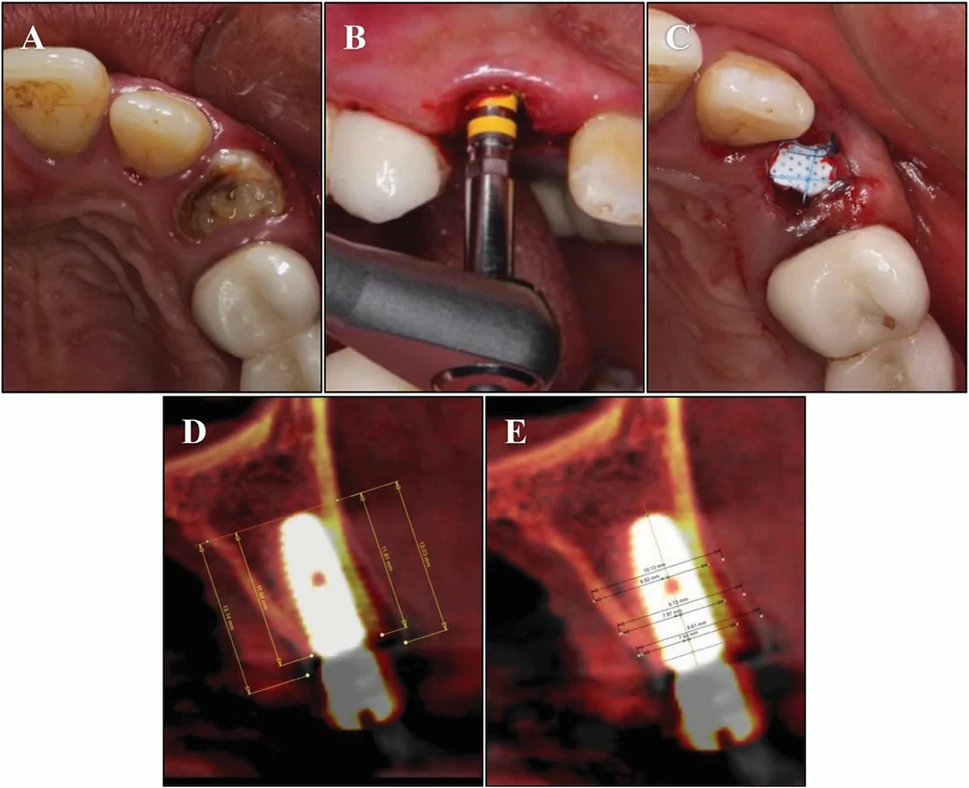
Control group; Preoperative occlusal view (**A**), Placement of the implant (**B**), Figure-eight suture over d-PTFE (**C**), Fused module of CBCT showing marginal bone loss (**D**), Fused module of CBCT showing reduction in bone width (**E**)

**Fig. 2:**
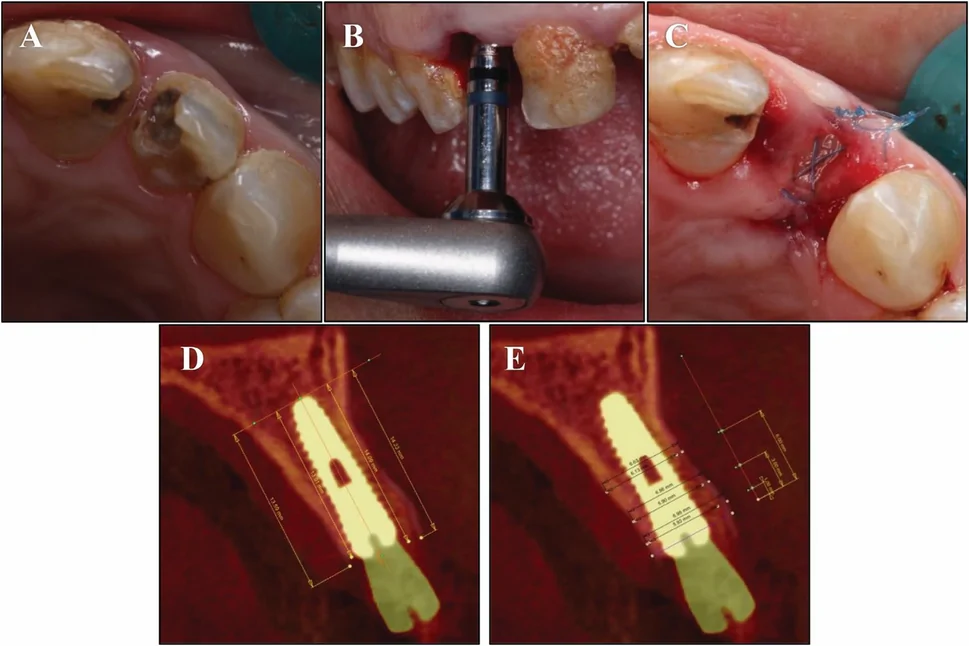
Study group; preoperative occlusal view (**A**), placement of the implant (**B**), figure-eight suture over ADM (**C**), fused module of CBCT showing marginal bone loss (**D**), fused module of CBCT showing reduction in bone width (**E**)

All patients underwent comprehensive medical and dental history assessments, followed by extraoral and intraoral examinations to confirm eligibility. Radiographic evaluation included orthopantomogram (OPG) for initial screening and cone beam computed tomography (CBCT) for baseline assessment and implant planning (Figs. 1A and 2A).

Patients were instructed to rinse with 0.12% chlorhexidine mouthwash (Orovex-H, Macro Group Pharmaceuticals, Egypt) for 1 min prior to surgery.

1. Peri-implant soft-tissue phenotype:

KMW was measured at baseline and at 6 months using a standardized periodontal probe. MT and STH were assessed at 6 months prior to prosthetic loading [19]. 

All phenotype measurements were recorded using a standardized periodontal probe: [19, 20]

- KMW was measured from the free mucosal margin to the mucogingival junction.
- MT was measured trans-mucosally perpendicular to the mucosal surface after local anesthetic administration, using the transgingival probing technique, with penetration thickness measured in millimeters until hard tissue contact.
- STH was measured as the vertical dimension of the soft tissue covering the implant. The measured distance was recorded in millimeters with the probe penetrating the peri-implant mucosa and positioned parallel to the implant’s long axis.

The 6-month soft-tissue assessment was performed after implant placement and before prosthetic loading.

2. Implant stability:

Implant stability was measured at placement (T0) and at 6 months (T1) using resonance frequency analysis (ISQ values) using a magnetic-based Osstell ISQ (Integration Diagnostics, Savadaled, Sweden) [21]. It was included as an objective, non-invasive measure of implant stability to evaluate the biological response to the two surgical procedures and to monitor changes in implant stability during the healing period.

3. Peri-implant clinical indices:

mSBI and PI were recorded at 1, 2, and 3 months following prosthetic loading. These indices were used as prespecified monitoring measures and were not intended to establish a contemporary diagnostic case definition of peri-implant health [22].

B. Radiographic evaluation

1. Marginal bone loss:

CBCT scans taken immediately after surgery (T_0_) were aligned with those obtained at the 6-month follow-up (T_1_). Marginal Bone Loss was assessed using a cross-sectional image registration technique (fused module) T_0_ and T_1_, allowing precise superimposition of the same anatomical site. The apical end of the implant as a reference point, with bone height measured on both the labial and palatal sides. Vertical bone loss was determined by subtracting the 6-month (T_1_) bone height from the immediate postsurgical measurement (T_0_), allowing for precise evaluation of bone changes [23].

2. Bone width:

Measured at multiple levels below the implant shoulder. Measurements were obtained using CBCT immediately after implant placement (T_0_) and after 6 months (T_1_). The T_0_ and T_1_ images were fused using a cross-sectional image registration technique (fused module), allowing precise superimposition and comparison of the same anatomical site over time. Measurements were taken at three specific heights (1 mm, 3 mm, and 6 mm below the implant shoulder) and the changes between T_0_ and T_1_were compared to assess alterations in horizontal bone dimensions [24]. 

#### Surgical procedure

All procedures were performed under local anesthesia using 4% articaine hydrochloride with epinephrine (1:100,000) (Inibsa Dental, Spain).

- Atraumatic extraction of the non-restorable maxillary anterior tooth was performed to preserve socket integrity. Osteotomy preparation was carried out using sequential drilling under copious sterile saline irrigation according to the manufacturer’s protocol. Implants (Titanium dental implants (CHAORUM Implant System, MEDIMECCA Co., Seoul, South Korea) were placed in a prosthetically driven position, achieving primary stability.

Primary implant stability was measured immediately after placement (T0) using resonance frequency analysis.

- Control group: The socket was covered with a d-PTFE (Cytoplast™, Osteogenics Biomedical, USA) membrane trimmed to fit the defect. The membrane was intentionally left exposed over the socket.
- (Study group): Limited split-thickness facial and palatal pouches were prepared, and an ADM allograft has thickness (mm) 0.75 – 1.25 and size (mm) 15–20 (OrACELL™, LifeNet Health, USA) was trimmed and inserted into the pouches to augment peri-implant soft tissues. The ADM is supplied fully pre-hydrated and ready-to-use without any additional preparation. It was handled aseptically according to the manufacturer’s instructions.
  No particulate bone graft was placed in either group.

In both groups, wound closure was achieved using figure-eight sutures with 6/0 polypropylene (Euro Surgical Limited, UK) (Figs. 1C and 2C).

#### Postoperative care

All patients received standardized postoperative instructions, including maintenance of oral hygiene and a soft diet for the first 3 days.

Antibiotics (amoxicillin–clavulanic acid 1 g; Augmentin^®^, GlaxoSmithKline, Egypt) were prescribed twice daily for 5 days, and analgesics (diclofenac potassium 50 mg; Cataflam^®^, Novartis, Egypt) once daily for 3 days. Patients were instructed to use 0.12% chlorhexidine mouthwash twice daily for 2 weeks starting from the second postoperative day.

Sutures were removed after 14 days. For group I, removal of the d-PTFE membrane did not require a second surgical procedure. The exposed membrane was simply grasped and removed after suture removal. It was removed without performing a secondary surgical procedure for retrieval.

A second-stage surgery was performed after 6 months for placement of healing abutments. Implant cover screw was accessed through a crestal incision, and this was performed in both groups.

This was followed by prosthetic rehabilitation using screw-retained zirconia restorations.

A. Clinical evaluation

### Statistical analysis

Data was analyzed using IBM-SPSS software (Statistical Package for Social Science) Version 27.0 (Armonk, NY: IBM Corp.). Qualitative data were presented as frequencies and percentages, while quantitative data were tested for normality using the Shapiro-Wilk test (p > 0.050 indicating normal distribution), Normally distributed quantitative data were expressed as mean ± standard deviation (SD), and non-normally distributed data as median and interquartile range (IQR). For comparisons, Chi-Square or Fisher’s exact test was used for qualitative variables, depending on expected cell counts. and the Independent-Samples t-Test or Mann-Whitney U test for quantitative variables. Associations between continuous variables were assessed using Pearson’s correlation for normally distributed data and Spearman’s correlation for non-normal or ordinal data. A p-value ≤ 0.050 will be considered statistically significant for all tests.

## Results

**Fig. 3:**
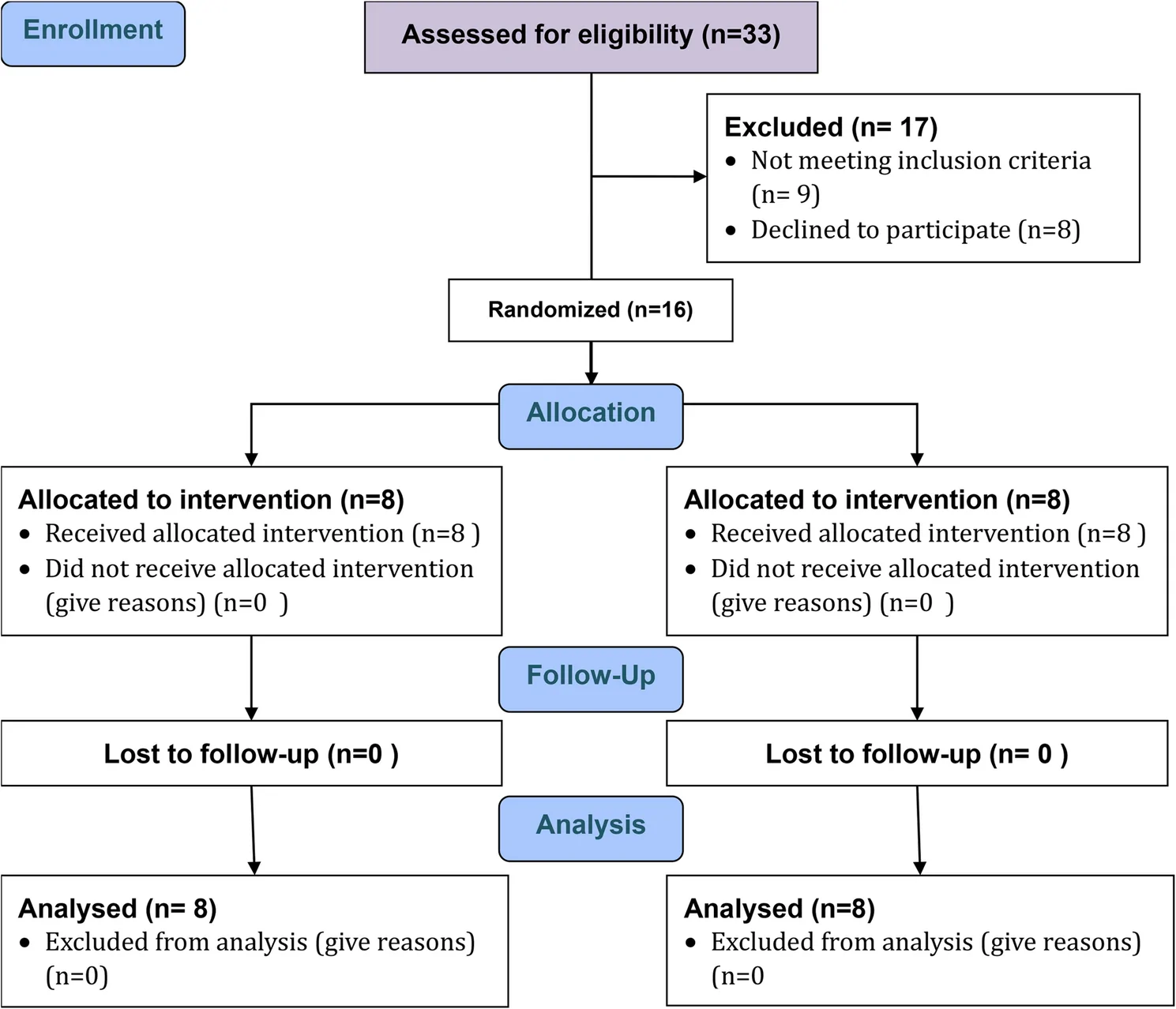
CONSORT diagram showing the flow of participants through each stage of randomized trial

Out of thirty-three patients assessed for eligibility, seventeen did not meet inclusion criteria and were excluded. Sixteen participants were randomized into two equal groups (*n* = 8 in Control group and *n* = 8 in study group. All allocated participants received the intended interventions, completed follow-up, and were included in the final analysis. The flow chart of the patients through the study followed the CONSORT flow diagram is presented in Figure 3.

### Demographic data and implant site

Baseline demographic characteristics were comparable between the two groups., the mean age of participants in control group was at 33.1 ± 6.4 years, study group was 31.7 ± 6.5 years (*p* = 0.676), Gender distribution showed 62.5% females (*n* = 5) in control group and 75% females (*n* = 6) in study group. Implant site was of equal split of upper canines, central incisors, and lateral incisors (25%, 37.5%, and 37.5% respectively, *p* = 1.000)(Table 1).

**Table 1:** Comparative analysis of demographic data and tooth position among the studied groups

Parameter		Control group *n* = 8 (%)	Study Group *n* = 8 (%)	*P*-value
Age (years)	Mean ± SD	33.1 ± 6.4	31.7 ± 6.5	0.676
Gender	Female	5 (62.5)	6 (75.0)	1.000
	Male	3 (37.5)	2 (25.0)	
Implant site	Upper Central incisor	3 (37.5)	3 (37.5)	1.000
	Upper lateral incisor	3 (37.5)	3 (37.5)	
	Upper canine	2 (25.0)	2 (25.0)	

SD: standard deviation Between-group comparisons for continuous variables (Age) used independent t-tests. Categorical variables (gender, Position) used fisher exact tests

*p* < 0.05 is statistically significant

### Clinical results

1. Soft tissue phenotype.

The interpretation of the findings to emphasize the observed treatment effects should rely on their 95% confidence intervals, rather than relying solely on statistical significance.

At baseline, KMW was 4.5 ± 0.8 mm in the control group and 5.2 ± 1.0 mm in the study group with no statistical significant difference (mean difference: 0.70 mm, 95% CI: -0.27 to 1.67, *p* = 0.143). However, at 6 months, KMW was 3.3 ± 0.6 mm in the control group and 5.9 ± 1.0 mm in the study group. This revealed a statistically significant increase in KMW in the study group (ADM) compared with the control group, with a mean difference of 2.60 mm (95% CI: 1.72–3.48; *p* < 0.001). Within group analysis showed a significant reduction in KMW in the control group (*p* = 0.003), whereas a significant increase was observed in the study group.

At 6 months, MT was 0.8 ± 0.3 mm and 1.8 ± 0.3 mm, with a mean difference of 1.00 mm (95% CI: 0.68 to 1.32, (*p* < 0.001) respectively, and STH was 0.8 ± 0.3 mm and 1.2 ± 0.3 mm, with a mean difference of 0.40 mm (95% CI: 0.08 to 0.72, (*p* = 0.012) respectively. These results demonstrated significantly greater MT and STH in the study group than in the control group (Table 2).

**Table 2:** Comparison of peri-implant soft tissue phenotype parameters between the control and study groups

Parameter	Time Point	(Control group) Mean ± SD	(Study group) Mean ± SD	Mean Difference (95% CI)	*P*-value
Keratinized mucosal width (mm)	Baseline (T0)	4.5 ± 0.8	5.2 ± 1.0	0.70 (-0.27, 1.67)	0.143
	6 months (T1)	3.3 ± 0.6	5.9 ± 1.0	2.60 (1.72, 3.48)	< 0.001*
	Within-group p-value	0.003*	< 0.001*		—
Mucosal thickness (mm)	6 months (T1)	0.8 ± 0.3	1.8 ± 0.3	1.00 (0.68, 1.32)	< 0.001*
Supracrestal tissue height (mm)	6 months (T1)	0.8 ± 0.3	1.2 ± 0.3	0.40 (0.08, 0.72)	0.012*

Data are presented as mean ± standard deviation (SD). T0 = baseline; T1 = 6 months postoperatively. Intergroup comparisons were performed using the independent samples t-test. Within-group comparisons for keratinized mucosal width were performed using the paired t-test

*Statistically significant at *p* < 0.05

2. Implant Stability:

Implant stability was comparable between groups at placement (T0) (*p* = 0.924). At 6 months (T1), the study group exhibited significantly higher implant stability than the control group (72.6 ± 2.1 vs. 69.0 ± 2.7 ISQ, respectively; *p* = 0.010). Both groups demonstrated significant increases in ISQ values from baseline to 6 months (*p* < 0.001) (Table 3).

**Table 3:** Comparison of implant stability between the studied groups at different time points

Time Point	(Control group)	(Study group)	
	Mean ± SD	Mean ± SD	*P*_1_value
T0 (Placement time)	59.6 ± 4.7	59.4 ± 5.6	0.924
T1 (6 Months)	69.0 ± 2.7	72.6 ± 2.1	**0.010**
P_2_value	**< 0.001**	**< 0.001**	

P_1_ value: Between-group comparisons used independent t-tests. P_2_ value: Within-group overall changes used paired t test

#### Modified sulcus bleeding index

No significant differences in mSBI scores were observed between the two groups at any follow-up time point (*p* > 0.05). Both groups showed low levels of peri-implant inflammation throughout the observation period, with median scores decreasing to 0 by the third month after loading (Table 4).

**Table 4:** Comparison of modified sulcus bleeding index mSBI between control group and study group

Time Point	Control group	Study group	
mSBI	Median (Range)	Median (Range)	*P*_1_value
T1 (1st month)	1 (0–2)	1 (0–1)	0.234
T2 (2nd month)	0 (0–2)	1 (0–1)	1.000
T3 (3rd month)	0 (0–2)	0 (0–1)	0.574
P_2_value	0.060	0.368	

P_1_ value: Between-group comparisons used Mann-Whitney U tests. P_2_ value: Within-group overall changes used Friedman tests

3. Plaque index:

Plaque index scores remained low throughout the follow-up period, with no significant differences detected between groups at any time point (*p* > 0.05). Both groups maintained excellent oral hygiene, as reflected by median PI scores of 0 during the study period (Table 5).

**Table 5:** Comparison of PI between the groups

Time Point	(Control group)	(Study group)	
PI	Median (Range)	Median (Range)	*P*_1_value
T1 (1st month)	0 (0–0)	0 (0–1)	0.442
T2 (2nd month)	0 (0–0)	0 (0–1)	0.442
T3 (3rd month)	0 (0–0)	0 (0–1)	0.721
P_2_value	1.000	0.368	

P_1_ value: Between-group comparisons used Mann-Whitney U tests. P_2_ value: Within-group overall changes used Friedman tests

### Radiographic results

#### Marginal bone loss

At 6 months, the study group demonstrated significantly lower marginal bone loss than the control group on both the buccal and palatal aspects. Median buccal bone loss was 0.35 mm in the study group compared with 1.87 mm in the control group (*p* < 0.001), while median palatal bone loss was 0.32 mm and 1.04 mm, respectively (*p* < 0.001) (Table 6).

**Table 6:** Comparison of MBL at 6 months between the studied Groups

Time Point	Control group	Study group	
MBL at 6 months	Median (Range)	Median (Range)	*P*-value
Buccal	1.87 (1.19–2.42)	0.35 (0.06–0.76)	**< 0.001**
Palatal	1.04 (0.8–1.54)	0.32 (0.16–0.75)	**< 0.001**

P value: Between-group comparisons used Mann-Whitney U tests

1. Bone width:

Both groups exhibited significant reductions in bone width from baseline to 6 months at all measured levels (*p* < 0.001). At 1 mm below the implant shoulder, bone width decreased from 8.0 ± 1.2 to 6.1 ± 0.8 mm in the control group and from 8.3 ± 0.9 to 7.0 ± 1.1 mm in the ADM group. At 3 mm, the corresponding values decreased from 8.1 ± 1.2 to 6.7 ± 0.7 mm and from 8.5 ± 1.2 to 7.2 ± 1.1 mm, respectively. At 6 mm, bone width decreased from 8.6 ± 1.0 to 7.6 ± 0.6 mm in the control group and from 8.8 ± 1.2 to 7.5 ± 1.1 mm in the ADM group. No statistically significant between-group differences were observed at any measurement level (Table 7).

**Table 7:** Comparison of bone width between the studied groups at different time points

Bone width	Time points	Control group	Study group	P_1_value
		Mean ± SD	Mean ± SD	
1 mm	T0	8.0 ± 1.2	8.3 ± 0.9	0.572
	T6	6.1 ± 0.8	7.0 ± 1.1	0.080
	*P*_*2*_ *value*	0.001	< 0.001	
3 mm	T0	8.1 ± 1.2	8.5 ± 1.2	0.568
	T6	6.7 ± 0.7	7.2 ± 1.1	0.253
	*P*_*2*_ *value*	< 0.001	< 0.001	
6 mm	T0	8.6 ± 1.0	8.8 ± 1.2	0.783
	T6	7.6 ± 0.6	7.5 ± 1.1	0.896
	*P*_*2*_ *value*	< 0.001	< 0.001	

P_1_value: Between-group comparisons used independent t-tests. P_2_value: overtime comparisons used paired t-test

*P* < 0.05 is statistically significant

## Discussion

Immediate implant placement in the anterior maxilla aims to optimize treatment efficiency and support favorable hard and soft tissue conditions for implant rehabilitation [25]. In the present study, all implants remained functional throughout the 6-month follow-up period, resulting in a survival rate of 100%. This finding is consistent with Happe, Schmidt and Neugebauer [26] demonstrating high survival rates for immediate implant placement protocols.

The clinical evaluation of peri-implant soft tissues demonstrated favorable outcomes in the ADM group, particularly regarding soft tissue thickness, keratinized mucosal width, and supracrestal tissue height. The mean mucosal thickness in the ADM group reached 1.8 mm at the 6-month assessment, while the control group exhibited significantly lower mucosal thickness values. These findings are consistent with those of Happe, Schmidt [17] who reported increased soft tissue thickness following the use of soft tissue grafting materials around immediate implant sites.

Similarly, the ADM group demonstrated significantly greater supracrestal tissue height and keratinized mucosal width compared with the control group. Adequate peri-implant soft tissue dimensions are considered essential for establishing and maintaining the peri-implant biologic seal and protecting the underlying crestal bone from mechanical and bacterial challenges. These findings are in agreement with those reported by Linkevicius, Puisys [27] and Quispe-López, Guadilla [19] who emphasized the importance of sufficient vertical and horizontal soft tissue dimensions in maintaining peri-implant tissue stability.

The direction of these findings is consistent with recent evidence showing that soft-tissue augmentation around implants can increase mucosal thickness and may support stability of the peri-implant mucosal margin. Nevertheless, current reviews emphasize heterogeneity among techniques and limited long-term evidence, particularly for ADM used with immediate implant placement; therefore, the present short-term findings should not be generalized beyond the studied protocol [6, 13]. 

Regarding the recorded peri-implant clinical indices, both groups exhibited low plaque accumulation and minimal bleeding scores throughout the loading period, with no significant differences between groups. These findings indicate low recorded plaque and bleeding values but do not constitute a contemporary diagnostic definition of peri-implant health. Similar observations have been reported by Stefanini, Barootchi [19] and Cucchi, Bettini [20] following soft-tissue augmentation and d-PTFE membrane applications.

Implant stability increased significantly in both groups during the healing period, reflecting successful osseointegration. Although primary stability was comparable at implant placement, the ADM group demonstrated significantly higher secondary stability at 6 months. This finding may be related to the enhanced soft tissue phenotype and reduced peri-implant bone remodeling observed in the ADM-treated sites. In contrast, Papi, Di Murro [28] reported less pronounced changes in implant stability following ridge preservation procedures using d-PTFE membranes.

Radiographic analysis revealed significantly lower marginal bone loss in the ADM group on both the buccal and palatal aspects. The median buccal bone loss in the ADM group was limited to 0.35 mm compared with 1.87 mm in the control group. Similarly, palatal bone loss was significantly lower in the ADM group. These findings support the concept that enhancement of the peri-implant soft tissue phenotype contributes to preservation of crestal bone levels. Linkevicius, Apse [29] previously demonstrated a strong association between increased peri-implant soft tissue thickness and reduced marginal bone remodeling.

Both groups exhibited significant reductions in horizontal bone width during healing, which is consistent with the physiologic remodeling process that follows tooth extraction and implant placement [30]. Although the ADM group showed a tendency toward greater preservation of bone width at the level closest to the implant shoulder, the difference did not reach statistical significance. Similar findings have been reported by Cosyn, Struys [31] who observed that soft tissue augmentation may reduce dimensional changes but does not completely prevent horizontal ridge remodeling. Therefore, while ADM appears beneficial in preserving peri-implant soft tissue dimensions and marginal bone levels, horizontal bone remodeling remains an inherent biological process following extraction and implant placement.

Our findings should be interpreted in the context of the well-documented physiological remodeling that occurs following tooth extraction. Experimental and clinical studies have demonstrated that immediate implant placement does not completely prevent post-extraction ridge remodeling, even when primary implant stability is achieved. Likewise, previous clinical investigations have highlighted the importance of peri-implant soft tissue management and careful case selection for optimizing esthetic and functional outcomes. The present findings are consistent with these observations and suggest that acellular dermal matrix may improve peri-implant soft tissue characteristics without implying complete preservation of the post-extraction alveolar ridge [32–34]. 

### Limitations

The present study has several limitations that should be considered when interpreting the findings. First, although the sample size was statistically sufficient to achieve adequate statistical power for our primary outcome, it may limit the generalizability of the results. Second, the study was conducted at a single center and included only patients requiring immediate implant placement in the maxillary esthetic zone. Future studies with larger sample sizes and longer follow-up periods are warranted to confirm the long-term benefits of ADM in immediate implant therapy.

## Conclusion

ADM was associated with improved peri-implant soft tissue phenotype and reduced marginal bone loss compared with d-PTFE membranes while maintaining comparable peri-implant health. These findings support the potential use of ADM during immediate implant placement in the anterior maxilla.

## Data Availability

The data sets used and/or analyzed during the current study are available from the corresponding author on reasonable request.

## Abbreviations

ADM: Acellular Dermal Matrix
d-PTFE: High-density Polytetrafluoroethylene
CBCT: Cone Beam Computed Tomography
KMW: Keratinized Mucosa Width
STH: Supracrestal Tissue Height
MT: Mucosal Thickness
mSBI / mBI: Modified Sulcus Bleeding Index
PI: Plaque Index
RFA: Resonance Frequency Analysis
ISQ: Implant Stability Quotient
BW: Bone width
MBL: Marginal Bone Loss
